# Effective deep learning aided vehicle classification approach using Seismic Data

**DOI:** 10.1038/s41598-025-01684-x

**Published:** 2025-07-02

**Authors:** Sherief Hashima, Mohamed H. Saad, Ahmad B. Ahmad, Takeshi Tsuji, Hamada Rizk

**Affiliations:** 1 Computational Learning Theory Team, RIKEN-Advanced Intelligence Project, Fukuoka, 819-0395 Japan; 2 Engineering Department, Nuclear Research Center, Egyptian Atomic Energy Authority, Cairo, 13759 Egypt; 3 Radiation Engineering Department, NCRRT, Egyptian Atomic Energy Authority, Cairo, Egypt; 4 School of Engineering, The University of Tokyo, 7-3-1 Hongo Bunkyo-ku, Tokyo, 113-8656 Japan; 5 Graduate School of Information Science and Technology, Osaka University, Suita, 565-0871 Japan; 6 RIKEN Center for Computational Science, Kobe, 650-0047 Japan; 7 Computer and Control Engineering Department, Tanta University, Tanta, 31733 Egypt

**Keywords:** Seismic signal, Vehicle classification, Intelligent transportation systems, Deep learning, Contrastive learning, Contrastive loss, Civil engineering, Electrical and electronic engineering, Computer science

## Abstract

Intelligent transportation systems (ITSs) significantly enhance traffic safety and management globally. A critical component of these systems is vehicle classification (VC), which supports vital applications such as congestion control, traffic monitoring, accident avoidance, etc. Traditional classification algorithms rely heavily on visual or sensor-based data (e.g., radar or image signals), often compromised by adverse weather, poor lighting, or occlusion. To address these limitations, this paper introduces a novel VC technique that leverages seismic data to detect vehicle-generated vibrations, thereby reducing susceptibility to environmental conditions and privacy concerns. We propose a self-supervised contrastive learning approach for seismic signal classification, eliminating the need for labeled data for feature extraction and representation. Our method employs specialized data augmentation techniques to create positive and negative pairs, enhancing feature representation. The encoder network extracts meaningful features from seismic signals while the projection head refines latent space representation. Training with contrastive loss ensures that positive pairs are closely aligned and negative pairs are distinctly separated in the latent space. Experimental results validate the efficacy of our approach, achieving state-of-the-art performance using seismic signal classification tasks with limited training data. Our approach achieves an impressive accuracy of 99.8%, underscoring its potential for robust and precise VC in ITSs using seismic data, particularly in data-scarce scenarios. The code is publicly available at https://github.com/MohamedHassanSaad/Vehicle-Classification.git.

## Introduction

**Fig. 1:**
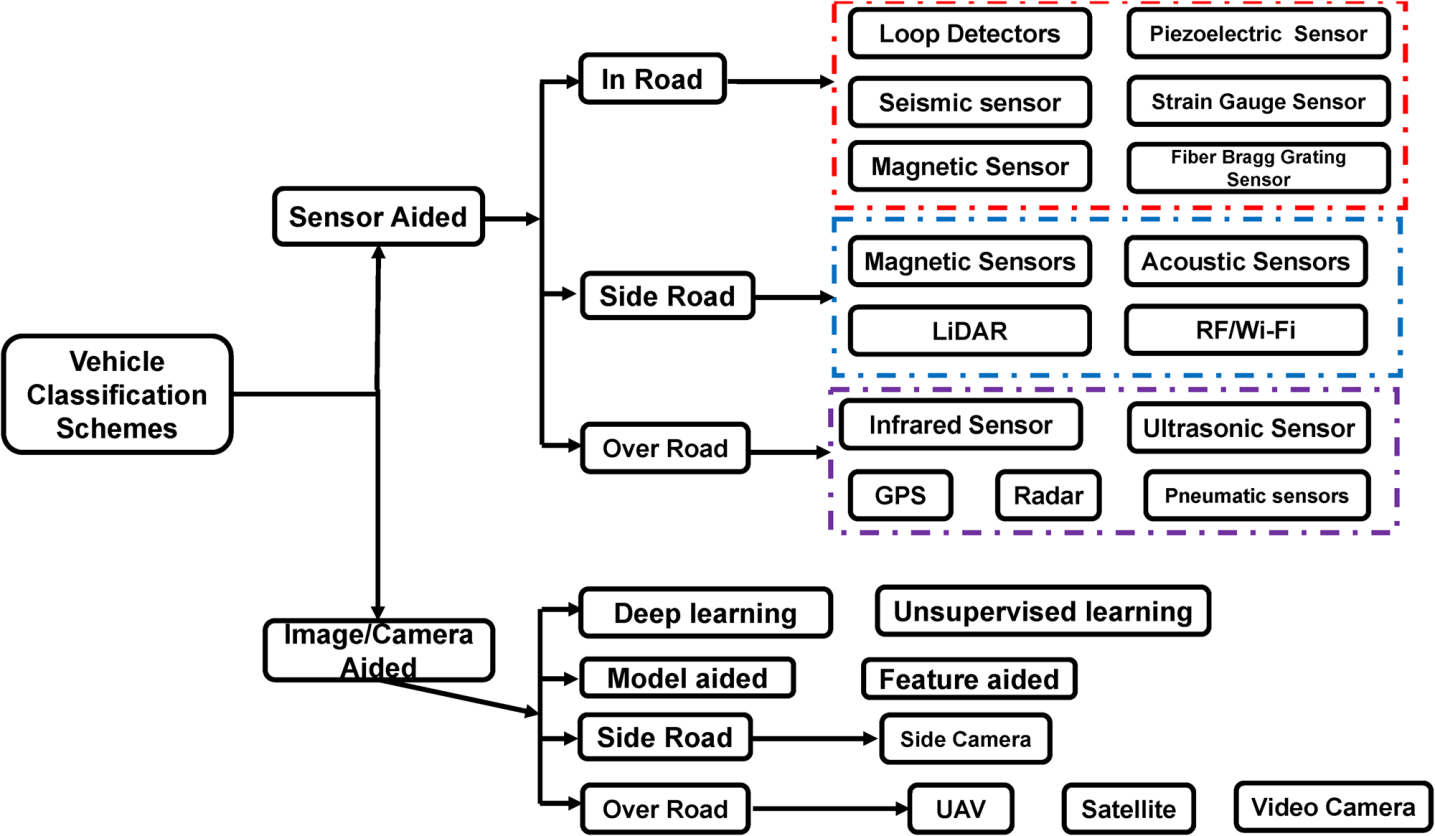
Vehicle classification schemes summary.

Due to its potential to support future exciting applications like autonomous driving, smart cities, etc., intelligent transportation systems (ITSs) have attracted a lot of research attention^1,2^. Vehicle classification (VC), which entails classifying cars into predetermined categories, is a crucial component of these systems^3^. Many applications require precise VC, including those from agencies that design and manage roads and highways. Developing and redesigning road infrastructure can be more effective when authorities know vehicle types, quantities, accident anticipation, and other characteristics^4,5^. Additionally, VC is essential for streamlining traffic, effectively distributing resources, and enhancing general road safety. These applications include toll collecting, autonomous driving, traffic flow management, automated parking, health monitoring, roadway monitoring, etc. Recent development of VC systems has been driven by notable developments in sensing and machine learning (ML) technologies, which have significantly improved classification accuracy and efficiency^6–8^. Nevertheless, these systems differ regarding features, needs, and operational conditions, including sensor types, parameter configurations, and financial implications. Generally, VC can be addressed using sensor-aided and image-based approaches^9–13^, as shown in Fig. 1.

The most popular camera-aided vision-based VC techniques have demonstrated remarkable classification accuracy 90–99%^3,14^. Camera-assisted VC covers large zones, making them ideal directions for broadband traffic surveillance activities. However, these systems are vulnerable to external factors, such as bad weather and difficult lighting conditions, making it challenging to detect cars that bigger ones hide. Furthermore, implementing such systems requires large expenditures on infrastructure during critical security and privacy issues^15^.

Inductive loop detectors, which use magnetic properties as an alternate solution, are now widely used in traffic monitoring systems to identify and categorize vehicles. These sensors use a wire coil placed under the road to record variations in electromagnetic profile signals, including amplitude, phase, and frequency, while cars drive over them^16^. Numerous investigations have repeatedly shown that loop detectors reach excellent levels of accuracy. Nevertheless, affordability and ease of installation impede the widespread adoption of loop detectors, mainly because coils must be embedded below the road’s surface. Given the inherent drawbacks and trade-offs of using loop detectors and traditional camera-based systems, our work aims to address these issues by employing seismic data collected by geophones.

Geophones are pivotal sensors that capture seismic signals, which are then processed to classify vehicular activities. These devices consist of a spring-mounted magnet moving within a coil, converting ground motion into electrical signals. The obtained signals are inherently noisy and require preprocessing to extract meaningful features. In our case, they are less vulnerable to external factors like weather and lighting, yet they yield insightful data about vehicle dynamics and features. We can identify unique characteristics and patterns in vehicle movement and vibration through seismic data analysis and overcome the limitations of Camera-based systems by employing geophones. Because road networks can strategically install geophones across them, seismic data can cover vast areas without requiring substantial infrastructure investments. The generated seismic data is valuable because it protects privacy and doesn’t record visual information about people or cars, unlike its typical camera-based competitors. However, the fluctuating and time-varying qualities of seismic data recorded by geophones make it challenging to extract significant patterns and characteristics.

Nevertheless, Seismic data presents unique challenges compared to traditional visual or sensor-based data, primarily due to its inherent noise and variability influenced by environmental factors, necessitating robust preprocessing techniques. Feature extraction from seismic signals requires specialized methods to transform raw data into a format suitable for classification, often involving advanced signal processing and domain-specific knowledge. The temporal dynamics of seismic signals add complexity to classification algorithms, as they must account for sequential data and potential temporal dependencies. Adequate training of deep learning models on seismic data also demands diverse and representative samples, achieved through complex augmentation techniques like time-shifting and noise injection. Additionally, leveraging self-supervised contrastive learning to eliminate the need for labeled data requires a carefully designed framework to ensure the model can learn from the inherent structure of seismic signals without explicit labels.

This paper presents a VC scheme within the realm of ITSs, utilizing seismic data acquired through geophones. This typical data source is characterized by its robustness in adverse environmental conditions and intrinsic privacy-preserving qualities, addressing privacy concerns. The study thoroughly investigates various data augmentation techniques tailored to seismic data, encompassing temporal manipulations to enrich the training dataset. A self-supervised contrastive learning framework is highlighted, designed to promote proximity among positive pairs (seismic waves from the same vehicle class) and segregation of negative pairs (seismic signals from different vehicle categories) within the latent space, optimized using the information noise-contrastive estimation (InfoNCE) loss function. The architecture of the encoder network includes 1D Convolutional Layers and a global average pooling (GAP) 1D Layer, along with the projection head, which is detailed later, and a classification head is added for VC after the contrastive learning phase. Our approach includes specialized noise reduction techniques and data augmentation strategies tailored to seismic data, ensuring robust feature extraction and classification. Seismic sensors have been effectively used in monitoring traffic flow, congestion control, detecting unauthorized vehicles in restricted areas, and even earthquake early warning systems, ensuring our approach’s robustness and precision in real-world scenarios. The paper validates the approach’s efficacy, demonstrating superior accuracy, particularly with the hybrid augmentation technique, even in scenarios of limited labeled data. Comparative evaluations against traditional classifiers consistently highlight the proposed method’s performance advantages. The paper underscores the practical relevance of the approach, emphasizing its accuracy and computational efficiency for applications in seismic analysis and related fields, offering a cost-effective and privacy-preserving solution without sacrificing performance.

To the best of our knowledge, we are the first pioneers to leverage contrastive learning for VC in ITS using seismic data with limited labeled data and semi-supervised learning. We have rigorously evaluated its effectiveness in high-noise environments and demonstrated its superior performance compared to existing techniques. The key contributions of this paper can be highlighted as follows:

- *Novel seismic-based vehicle classification* : We introduce a pioneering approach that leverages seismic data collected through geophones for VC within ITSs. This method is robust in adverse environmental conditions and preserves data privacy.
- *Self-supervised contrastive learning framework: * We propose a self-supervised contrastive learning framework for classifying seismic signals, which operates without needing labeled data for feature extraction and representation. This approach enhances feature representation and achieves high accuracy, even with limited labeled data.
- *Comprehensive data augmentation techniques: * We explore various data augmentation techniques tailored to seismic data, including time shifting, time reversal, sample down-sampling, sample up-sampling, and hybrid augmentation. These techniques enrich the training dataset and improve model robustness.
- *Evaluations: * We validate the efficacy of the proposed approach through a robust empirical validation process. The paper performs comparison analyses using conventional classifiers, which include logistic regression, naive Bayes (NB), support vector machines (SVM), convolutional neural networks (CNN), and long-term short-term memory (LSTM). The results consistently highlight the proposed methodology’s performance benefits.

Our proposed approach reduces costs and protects privacy without sacrificing performance, which makes it more appealing and functional in real-world applications, such as traffic management, improved safety with quicker accident detection, cost-effective and reliable solutions unaffected by weather conditions, privacy-preserving highway toll gates, controlling road lanes (emergency lanes), intelligent infrastructure maintenance, environmental monitoring, and heightened security by detecting unauthorized vehicles.

The remainder of the paper flows as follows: The related work is summarized in Section “Related work”. Our entailed VC system model is detailed in “VC system overview”. Furthermore, Section “Proposed contrastive learning method” highlights our proposed contrastive learning approach for VC. Evaluation results and findings are detailed in Section “Results and discussion”. Finally, the completed thoughts and future directions are carried out in Section “Conclusion and future directions”.

## Related work

Lately, burgeoning research has focused on harnessing VC approaches to develop more intelligent ITSs.^1,3^ presented an extensive survey covering various VC techniques for ITSs. Recent research highlights significant challenges related to data privacy and the reliance on extensive training datasets. While methods like those in^17–19^ utilize AI and IoT to enhance transportation safety and efficiency, they face privacy concerns and infrastructure requirements due to their dependence on image data. Approaches such as^20,21^ incorporate deep learning techniques but still require large, labeled datasets, raising scalability and privacy issues. However, methods like^22^, which introduce active learning frameworks, aim to address the need for smaller datasets and improved accuracy. Additionally,^23^ extends ego-vehicle perception through non-visual methods, reducing privacy concerns, while^24,25^ have leveraged advanced CNN techniques to minimize computational demands. Despite these advancements, privacy issues and dataset dependency remain ongoing challenges, with many methods still needed to balance efficiency and data sensitivity.

Nevertheless, although the above-stated techniques are helpful, they frequently disregard privacy concerns and necessitate big image datasets for testing and training. Another study area explores DL techniques for VC using temporal seismic data, as seen in^26–29^. These methods show promising solutions for reducing data requirements and enhancing privacy but are still in the early stages of development. The work of^30^ proposed a deep CNN architecture combined with a log-scaled frequency cepstral coefficient (LFCC) matrix to classify vehicles using seismic signals. However, their solution needs more improvements. Furthermore, the SenseMag method introduced in^16^ makes use of two noninvasive magnetic sensors that are placed strategically along road sections. Surprisingly, the trials on Chinese highways produced an astounding VC accuracy of 90%. However, this approach requires specific sensor placements and may not be scalable. The work of^31^ practically developed an innovative, flexible magnetometer sensor to count and classify vehicles with promising classification capabilities. While effective, this method requires specialized sensors and installation. Another classification idea depends on investigating the WIFI channel state information of the moving vehicles^32^. This approach requires extensive infrastructure and is susceptible to interference. Besides, acoustic sensors can be distributed using fiber optic cables for intelligent traffic monitoring by transforming telecommunications cables into seismic sensors as in^33^. Still, these techniques suffer from the need for an extensive infrastructure, complicated installation techniques, and susceptibility to damage.

The work of^34^ applied contrastive learning for impulse radio ultrawideband (IR-UWB) radar for VC. While this approach shows promise in improving VC accuracy, it requires specialized radar equipment, which can be costly and complex to deploy at scale. Also, a contrastive learning-aided approach was used to effectively classify Synthetic aperture radar (SAR) images in^35^. Although effective, this method relies on high-resolution SAR images, which can be expensive to obtain and process and may not be feasible for real-time applications. The authors of^36^ applied supervised contrastive learning (ResNet) and transfer learning techniques for vehicle intrusion systems to prevent car hacking. Nevertheless, this approach requires extensive labeled data for training, which can be challenging to acquire and maintain. Furthermore, a semi-supervised Contrastive Learning approach was proposed in^37^ to aid in autonomous vehicle driving via proper video-to-video distances known as ego vehicle actions. While this method reduces the need for labeled data, it still requires significant computational resources. Furthermore, the work of^38^ introduced a multi-view graph contrastive learning (MVGCL) method to handle uniform vehicle routing problems (VRPs). Although innovative, this approach relies on complex graph structures and may require substantial computational power, making it less practical for large-scale deployment. A self-supervised bidirectional trajectory contrastive learning (BTCL) model for driving intention prediction was proposed in^39^ with an excellent ability to learn high-quality trajectory representations without labeled data. Still, our proposed method addresses privacy concerns, reduces data requirements, and offers a cost-effective solution without sacrificing performance.

## VC system overview

**Fig. 2:**
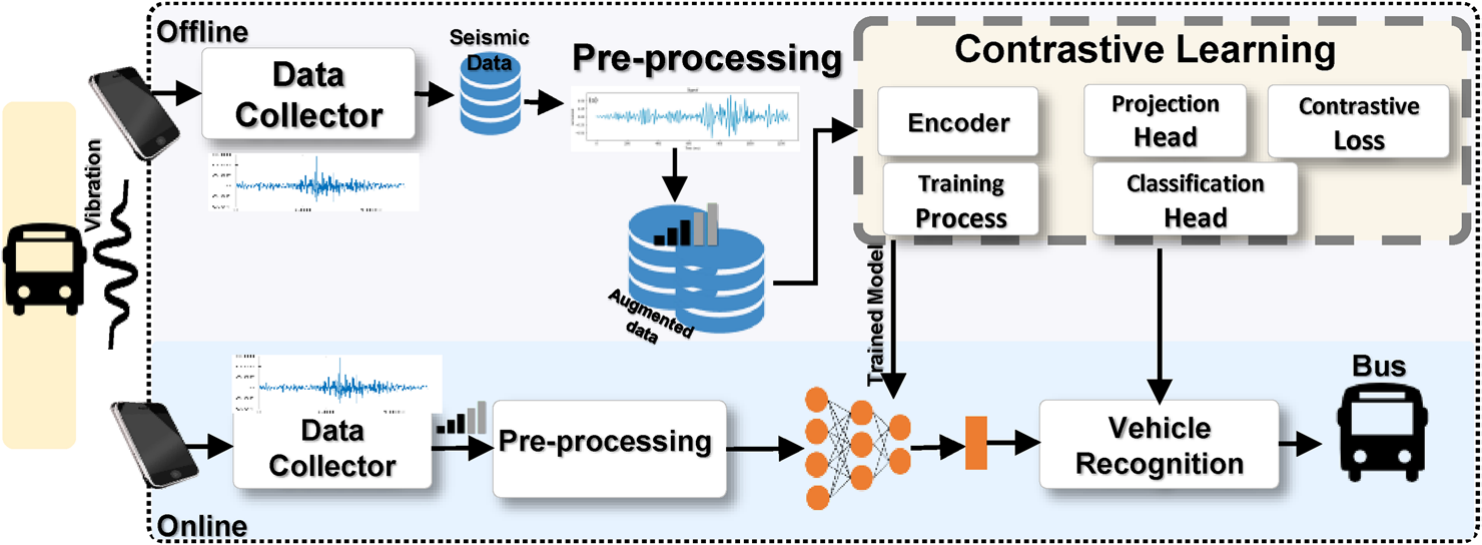
Proposed contrastive learning aided VC technique.

Figure 2 presents the main components of the proposed VC system model. The following subsections describe each model in detail.

### Data collection

**Fig. 3:**
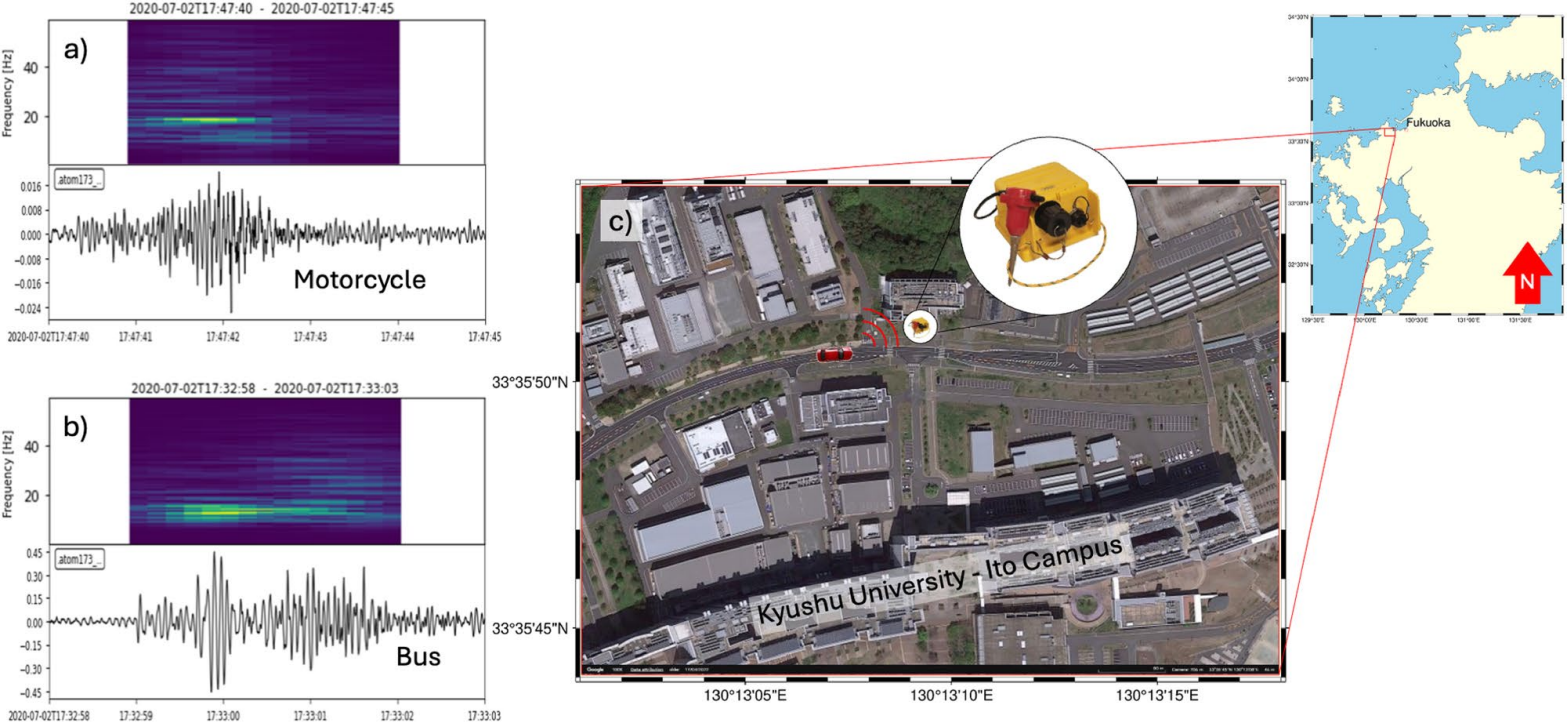
Seismic signals and spectrograms of a (**a**) motorcycle and (**b**) bus recorded by a sensor. (**c**) Sensor location at Kyushu University Ito Campus, Fukuoka, Japan, shown using a satellite image from Google Earth^40^ ($\copyright$ Google, Image Landsat/Copernicus), prepared and annotated with PyGMT (v0.14.2, https://www.pygmt.org).

Herein, geophones were used to gather seismic data from passing vehicles at Kyushu University, Japan in July 2020, as shown in Fig. 3. The geophones were positioned at three stations, each 15 meters apart and located 0.5 meters from the road, capturing vertical vibrations at a sampling rate of 250 Hz. The vehicles were categorized into three groups by size: large (such as buses and trucks), medium (private cars), and small (motorcycles and scooters). Vehicle speeds ranging from 25 to 35 km/h with a maximum of 45 km/h were estimated using seismic signals from the three stations (Herein, road regulations limit vehicle speeds to 40 km/h). A video camera provided visual guidance for manually preparing the training dataset only clear signals from vehicle events were chosen to avoid model overfitting, excluding those with noise or overlapping vehicle signals. The selected events were converted into 5-s windows with a 250 Hz sampling rate, ensuring the inclusion of the entire seismic waveform. In total, 600 waveforms were created from the three vehicle categories. Each category contained 200 waveforms, while an additional 300 windows representing noise from various sources (e.g., wind, pedestrians, road work) were included. Augmented data were only used in the training phase while testing used original unseen data.

**Fig. 4:**
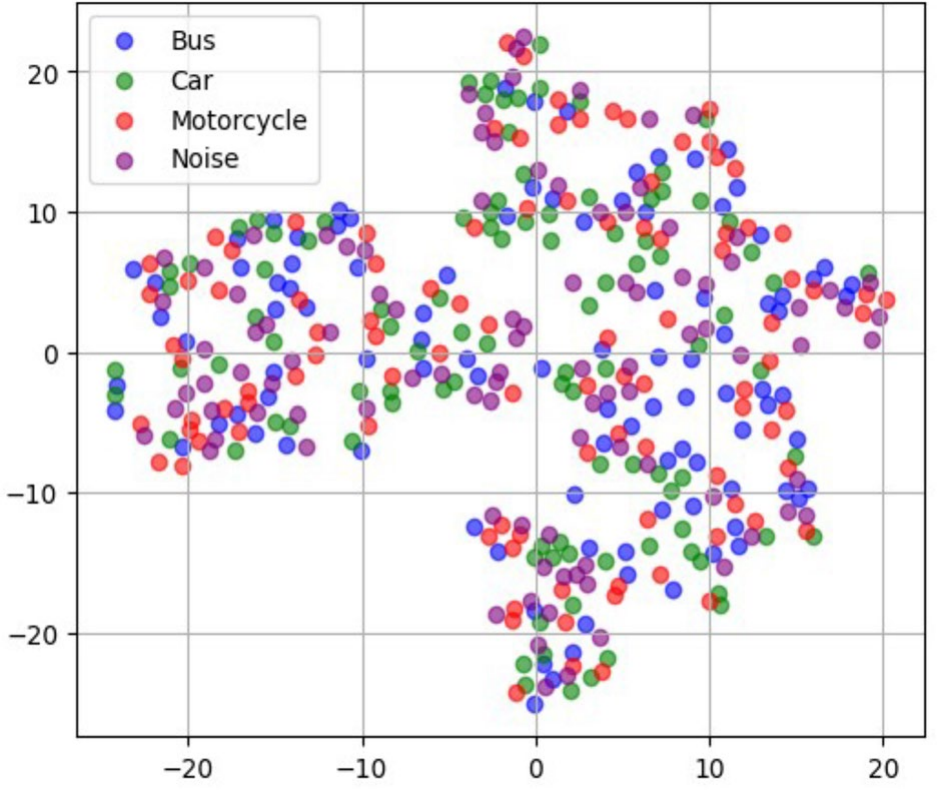
t-SNE visulization of raw data.

Using geophones facilitated precise seismic activity measurement, providing high-resolution records of ground motions in the road environment. These data offer valuable insights into vehicle characteristics and dynamics for further analysis and classification. Figure 4 shows the t-SNE visualization of the collected seismic data, which reveals overlapping clusters for different vehicle types and highlights the potential challenge in classification and the need for further feature extraction.

### Data preprocessing

**Fig. 5:**
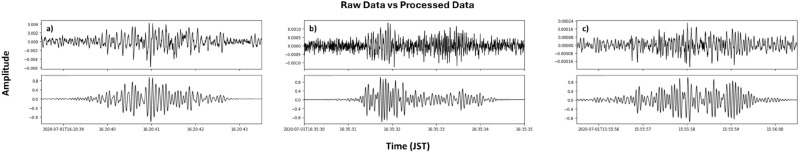
Collected Seismic Data of (**a**) Bus (**b**) Car (**c**) Motorcycle (Top: Raw data & Bottom: Processed Data).

Data pre-processing is crucial for enhancing the quality and dependability of seismic data by ensuring it is clean, normalized, and properly prepared for further analysis. Figure 5 illustrates the data before and after preprocessing to decrease noise for different vehicle types (bus, car, and motorcycle). Seismic data naturally exhibits amplitude variations due to factors like the distance from the source and the sensitivity of the receiver. To mitigate these effects, we applied a band-pass filter with a frequency range of 5–35 Hz to remove unwanted low-frequency noise and high-frequency interference. Additionally, a Hamming window was used to minimize spectral leakage and enhance signal clarity. Finally, min-max normalization was performed to scale the seismic data to a standardized range between 0 and 1, as demonstrated in the following equation:

1 $$\begin{aligned} x^{\prime }=\frac{x-\min (x)}{\max (x)-\min (x)} \end{aligned}$$

Here, *x* represents the original seismic data, while $x^{\prime }$ denotes the normalized version. Using the min-max normalization technique, the seismic data is scaled to a uniform range between 0 and 1. This normalization process improves data compatibility across various sources and receivers, facilitating more precise and insightful analysis.

### Data augmentation

Seismic signals exhibit considerable variability due to external factors such as environmental noise, road surface conditions, and sensor sensitivity. This variability poses a significant challenge for vehicle classification, as similar vehicles may produce slightly different signals under varying conditions. To address this, we incorporate a different data augmentation strategy that enhances the contrastive learning framework, improves feature extraction, and promotes class separability in the latent space. Augmentation is a crucial component in contrastive learning, as it allows the model to generate multiple representations of the same underlying signal while maintaining the essential characteristics that define the vehicle class. This enables the model to learn robust representations of minor perturbations and domain shifts, ultimately improving classification accuracy.

The augmentation process involves generating positive pairs by applying transformation techniques to seismic signals while ensuring that the fundamental characteristics of the waveform remain intact. This is particularly important in contrastive learning, where the model is trained to minimize the distance between positive pairs in the latent space while maximizing the separation between negative pairs. Without augmentation, the learned feature space may become overly dependent on the specific characteristics of individual signals, limiting the generalization capability of the model. To prevent this, we leverage a suite of augmentation techniques, including time shifting, time reversal, down-sampling, up-sampling, and hybrid augmentation, each designed to enhance the diversity of training samples while preserving the critical structural patterns in the seismic data.

**Fig. 6:**
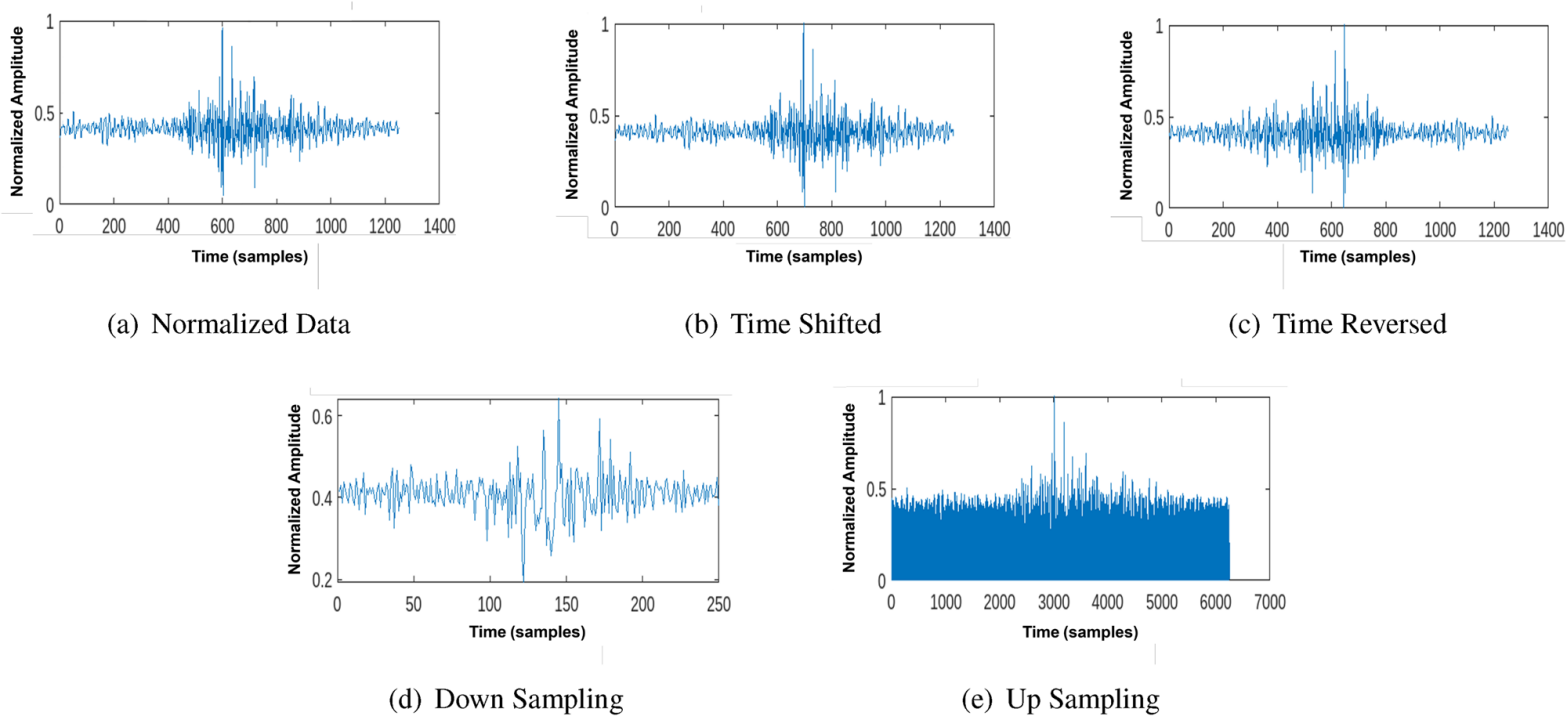
Example of augmentation techniques applied to a normalized seismic signal from a bus.

- *Time shifting:* modifies the temporal alignment of the seismic signal by shifting the waveform forward or backward along the time axis, as shown in Fig. 6b. This transformation simulates real-world variations caused by differences in vehicle speed, sensor placement, or slight inconsistencies in recording timestamps. By applying time shifts, the model learns to recognize the core structural patterns of the signal rather than relying on the absolute positioning of peaks and troughs in the waveform. This ensures that classification performance remains unaffected by minor temporal misalignments. However, excessive time shifting can distort the relationship between key signal components, making it necessary to optimize the shift magnitude to preserve essential signal characteristics.
- *Time reversal:* flips the order of the waveform, effectively generating a mirrored version of the seismic signal. Unlike time shifting, which preserves the original signal sequence, time reversal alters the directionality of temporal features while retaining the overall spectral composition, as shown in Fig. 6c. This augmentation forces the model to learn directionally invariant representations, particularly useful in seismic signal processing, where symmetrical waveforms often arise due to reflections from the road surface or underlying structures. By training on both original and reversed waveforms, the model gains the ability to recognize vehicles based on frequency and amplitude patterns rather than strict temporal ordering, improving generalization across diverse signal conditions.
- *Down-sampling:* reduces the resolution of the seismic signal by selectively removing data points, as shown in Fig. 6d. This transformation forces the model to extract coarser, high-level features that remain stable across different resolutions. By reducing the dependency on high-frequency variations, down-sampling improves the model’s ability to focus on global signal patterns rather than overfitting to fine-grained noise. This is particularly useful in environments where sensor quality or data transmission rates may vary.
- *Up-sampling:* Conversely, it increases the temporal resolution of the signal by interpolating additional data points. This augmentation ensures that important waveform structures are preserved even when signals are subjected to compression or lower sampling rates, as shown in Fig. 6e. By exposing the model to both down-sampled and up-sampled versions of the data, we improve its ability to handle real-world variations in seismic recordings.
- *Hybrid augmentation:* combines multiple augmentation techniques to introduce higher-order variations while preserving essential class-defining features. Unlike individual augmentations, which apply a single transformation simultaneously, hybrid augmentation leverages complementary perturbations in sequence. For instance, time shifting followed by down-sampling ensures that the model remains invariant to both temporal misalignments and resolution variations. In contrast, time reversal followed by up-sampling enhances directional robustness while preserving fine-grained details. Hybrid augmentation maximizes intra-class diversity while maintaining inter-class discrimination, leading to a more structured latent space that facilitates improved classification.

**Fig. 7:**
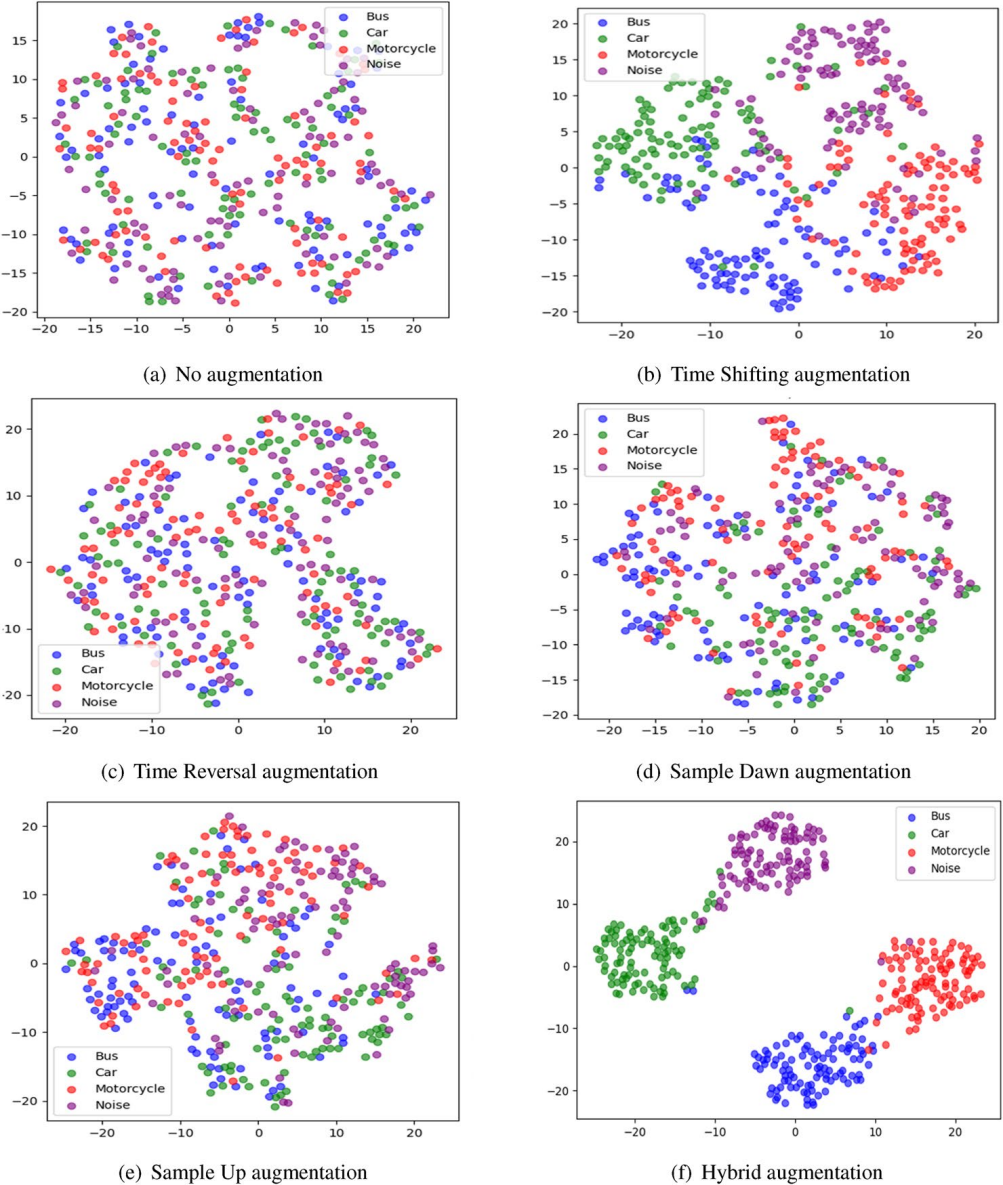
t-SNE visualization of different augmentation techniques.

The effectiveness of data augmentation in improving class separability is demonstrated in Fig. 7, which presents t-SNE visualizations of the learned feature space under different augmentation schemes. Without augmentation (Fig. 7a), the feature clusters exhibit significant overlap, indicating that raw seismic signals alone do not provide sufficient discriminatory power for vehicle classification. When applying individual augmentations, such as time shifting or time reversal (Fig. 7b–e), some degree of separation is observed, but the clusters remain partially entangled. However, when hybrid augmentation is employed (Fig. 7f), the clusters become more distinct, with clear boundaries between vehicle classes. This highlights the ability of augmentation to reduce intra-class variance while maximizing inter-class separation, an essential characteristic for effective contrastive learning.

Beyond improving class separability, data augmentation plays a crucial role in feature extraction by exposing the model to multiple transformations of the same sample. This encourages the encoder network (in Subsection “Encoder network”) to learn domain-invariant features that remain stable across different conditions. These refined features are further optimized by the contrastive loss function (described in Subsection “Contrastive loss”), which ensures that signals from the same vehicle class remain close in the latent space while signals from different classes are pushed apart. The result is a well-structured feature space that enables high-accuracy classification, even in low-data or high-noise environments.

## Proposed contrastive learning method

This section introduces the self-supervised contrastive learning framework proposed for seismic-based vehicle classification. The rationale behind adopting contrastive learning stems from the inherent challenges of seismic signal classification. Seismic signals exhibit high intra-class variance due to environmental noise, road surface variations, and sensor positioning, making feature extraction challenging. Traditional supervised learning methods, such as convolutional neural networks (CNNs) and recurrent neural networks (RNNs), require extensive labeled data to learn class-specific patterns. However, annotating seismic signals is costly and impractical, particularly in large-scale intelligent transportation systems. Our self-supervised contrastive learning framework overcomes these limitations by leveraging unlabeled seismic signals to pre-train the model, which is later fine-tuned on a small labeled subset for classification.

Compared to supervised learning, our approach offers several key advantages. First, it eliminates the need for large labeled datasets by learning feature representations in an unsupervised manner. Second, it improves class separability by structuring the learned representations in a way that maximizes intra-class similarity while maintaining inter-class separation. Third, it enhances model robustness to environmental variations, as the augmentation-based contrastive training ensures that the model generalizes well across diverse conditions. The effectiveness of this approach is validated through t-SNE visualizations (see Fig. 9) and empirical evaluations, demonstrating that contrastive learning significantly outperforms traditional classifiers in terms of feature discrimination and classification accuracy.

**Fig. 8:**
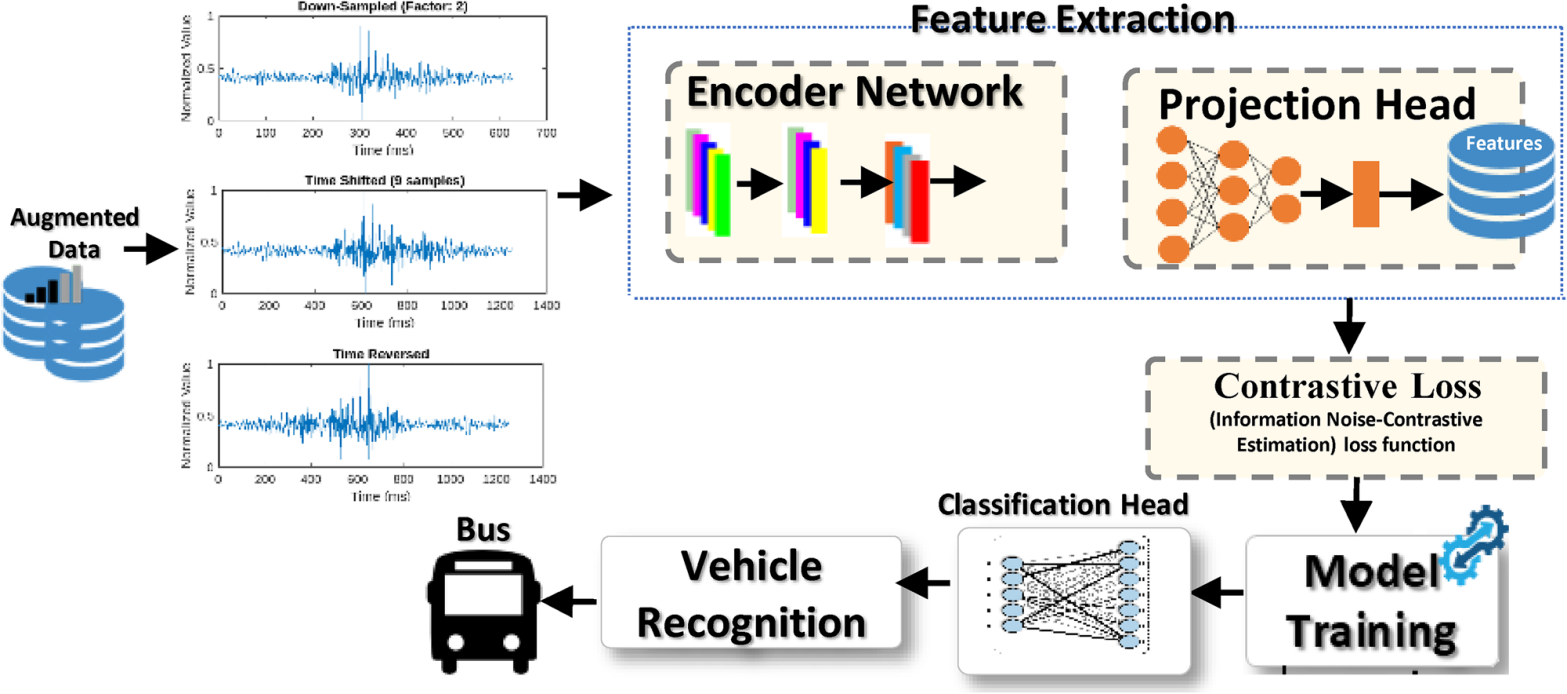
Contrastive learning-based network architecture for VC.

The proposed technique consists of three primary components (see Fig. 8): (1) an encoder network that extracts meaningful features from seismic signals, (2) a projection head that refines these features in a contrastive learning space, and (3) a contrastive loss function that optimizes the model by ensuring that similar signals are mapped closer together while dissimilar signals remain well-separated.

**Fig. 9:**
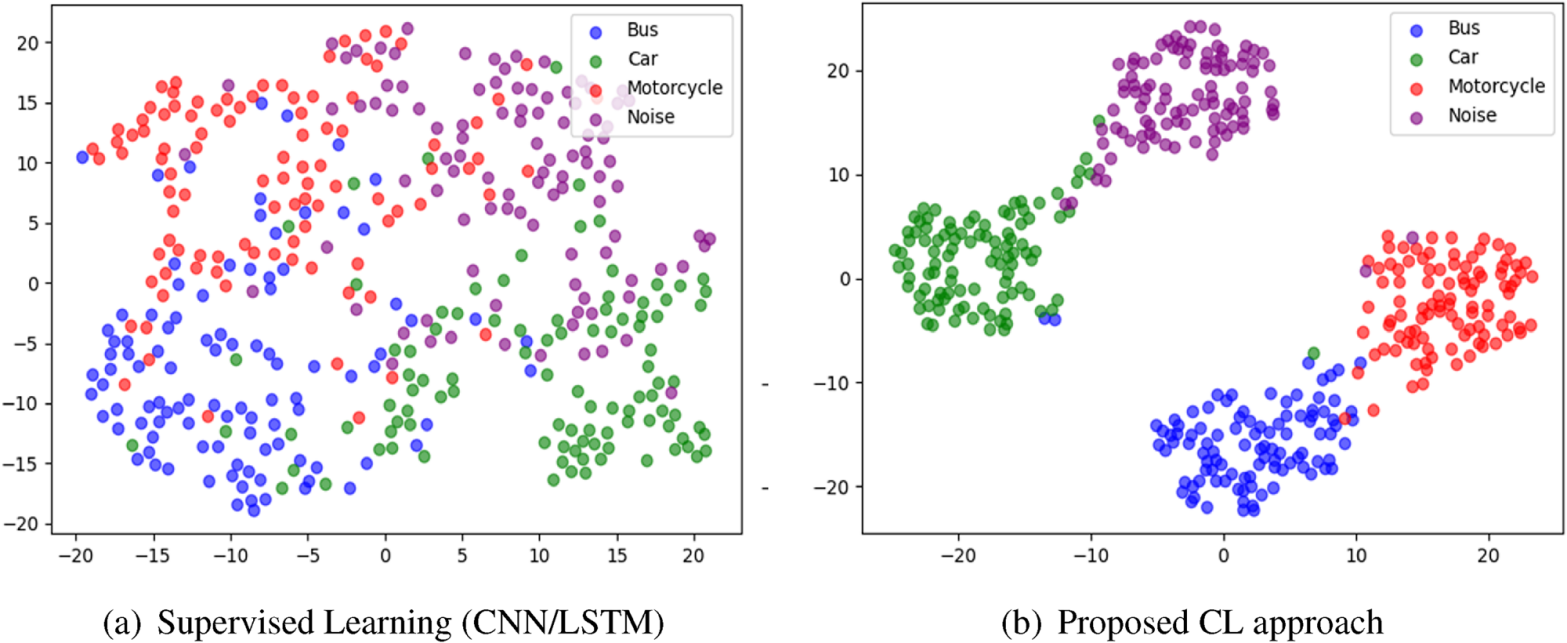
Comparison between the supervised learning approach vs the proposed CL using t-SNE visualization.

The network architecture consists of two 1D Convolutional Layers followed by ReLU activations, a global average pooling 1D Layer for feature extraction, and a Dense layer with ReLU activation for projection into a compact latent space. Contrastive loss, specifically InfoNCE loss, encourages positive pairs of seismic waves from the same class to be closer and negative pairs from different classes farther apart in the latent space. By optimizing the contrastive loss during training, the model learns to extract meaningful and discriminative features from seismic signals (as shown in Fig. 9), leading to state-of-the-art performance on classification tasks. The model is further fine-tuned on labeled data to adapt the learned features for the specific classification task, yielding accurate and efficient seismic signal classification. Table 1 summarizes the architecture of the proposed CL model.

**Table 1:** Summary of the proposed contrastive learning model.

Layer type	Output shape	Parameters
Input layer	(128, 128, 1)	0
Conv2D (64 filters, $3\times 3$)	(126, 126, 64)	640
BatchNorm2D	(126, 126, 64)	256
ReLU activation	(126, 126, 64)	0
MaxPooling2D ($2\times 2$)	(63, 63, 64)	0
Conv2D (128 filters, $3\times 3$)	(61, 61, 128)	73,856
BatchNorm2D	(61, 61, 128)	512
ReLU activation	(61, 61, 128)	0
MaxPooling2D ($2\times 2$)	(30, 30, 128)	0
Conv2D (256 filters, $3\times 3$)	(28, 28, 256)	295,168
BatchNorm2D	(28, 28, 256)	1,024
ReLU activation	(28, 28, 256)	0
MaxPooling2D ($2\times 2$)	(14, 14, 256)	0
Flatten	(50176)	0
Dense (512 units)	(512)	25,690,624
Dropout (0.2)	(512)	0
Dense (128 units)	(128)	65,664
Dense (feature embedding)	(128)	16,512
Contrastive loss layer	(128)	0

### Encoder network

The encoder network extracts meaningful features from the seismic signal data. It is designed to capture low-level and high-level patterns and correlations essential for accurate classification. The architecture of the encoder network comprises the following components:

- Two 1D Convolutional Layers: The first Conv1D layer performs a 1D convolution operation with 32 filters and a kernel size of three. It focuses on detecting local patterns and low-level features in the input seismic signal. The second Conv1D layer follows with 64 filters and a kernel size of 3, capturing higher-level features that represent more abstract patterns in the signal.
- ReLU Activation Function: After each convolutional layer, we apply the ReLU activation function element-wise. ReLU introduces non-linearity to the network, allowing it to more effectively capture complex relationships and representations in the data.
- GlobalAveragePooling1D Layer: This layer pools the spatial information in the output of the second Conv1D layer and computes the average value across all data points. The resulting fixed-length feature vector represents each seismic sample’s condensed and informative representation, regardless of its original length. This step enables the model to handle variable-length input signals efficiently during classification.

### Projection head

The projection head is an additional component that takes the extracted features from the encoder and maps them into a more informative and compact latent space representation. This step aims to enhance the discriminative power of the learned features and facilitate better clustering of similar samples. The projection head consists of a Dense layer with 128 units and a ReLU activation function.

- Dense Layer: The dense layer transforms the extracted features from the encoder network and projects them into a more meaningful and condensed representation.
- ReLU Activation Function: We apply the ReLU activation function after the Dense layer to introduce non-linearity, enabling the model to capture complex and non-linear relationships in the latent space.

The encoder network comprises two 1D convolutional layers followed by a global average pooling (GAP) layer, allowing it to capture fine-grained local patterns and global temporal structures within seismic signals. The extracted feature representations are then passed through the projection head, a dense layer with ReLU activation. It refines and maps them into a lower-dimensional space optimized for contrastive learning. This design enhances feature separability, reducing intra-class variance while maximizing inter-class differences. To validate the effectiveness of this architecture, t-SNE visualizations of the latent space of the proposed method in Fig. 9b demonstrate that seismic signals from the same vehicle category form well-clustered groups while different vehicle types are distinctly separated. Additionally, the low contrastive loss values (0.010–0.015) observed during training indicate that the learned feature representations effectively discriminate between vehicle classes. The combination of the encoder and projection head ensures the robustness of the self-supervised contrastive learning framework, improving classification performance even in high-noise environments with limited labeled data.

### Contrastive loss

The contrastive loss is a key component of our self-supervised contrastive learning approach. Its objective is to encourage positive pairs closer in the latent space while pushing negative pairs further apart. We use the InfoNCE (information noise-contrastive estimation) loss function. Positive pairs are formed by pairing seismic waves from the same class, while negative pairs consist of seismic waves from different classes. The InfoNCE loss compares the cosine similarity between anchor-positive and anchor-negative pairs, encouraging positive pairs to have higher similarity than negative pairs. By minimizing this loss, the model learns to create meaningful clusters of similar samples in the latent space, enabling effective feature extraction.

The InfoNCE loss for a single anchor-positive pair and a set of negative pairs can be written as:

2 $$\begin{aligned} \text {InfoNCE Loss} = -\log \left( \frac{\exp \left( \frac{\text {sim}(v_i, v_i^+)}{\tau }\right) }{\sum _{j=1}^{N} \exp \left( \frac{\text {sim}(v_i, v_j^-)}{\tau }\right) } \right) , \end{aligned}$$

where $v_i$, $v_i^+$ are embedding an anchor seismic wave and positive pair seismic wave (from the same class), respectively. $v_j^-$ refers to the embedding of a negative pair seismic wave (from a different class), ${\text {sim}(v_i, v_i^+)}$ represent the cosine similarity between vectors, ${\tau }$ defines the temperature parameter controlling the smoothness of the distribution, and *N*is the number of negative pairs considered for each anchor-positive pair.

The contrastive learning approach ensures the model captures relevant patterns and correlations in the seismic data, leading to superior classification performance. The model acquires a rich latent space representation that effectively clusters similar seismic signals and maintains clear separations between different classes by optimizing the contrastive loss. By optimizing the contrastive loss during training, the model learns to extract relevant and discriminative features from the seismic signal data, ultimately leading to improved classification performance.

### Model training

Positive and negative pairs are iteratively fed through the encoder network during training. The contrastive loss is calculated for each pair, and the model’s parameters are updated using backpropagation to minimize the loss. The model learns to generate meaningful and compact representations for the seismic data by repeating this process for multiple epochs.

The objective function, representing the contrastive loss, is given by:

3 $$\begin{aligned} \mathscr {J} = \frac{1}{M} \sum _{i=1}^{M} \frac{1}{K} \sum _{j=1}^{K} L(i, j, k), \end{aligned}$$

where *L*(*i*, *j*, *k*) is the contrastive loss for a pair (*i*, *j*, *k*), *M* is the total number of seismic samples, and *K* is the number of positive pairs for each original seismic sample.

The optimization step involves updating the model parameters through gradient descent:

4 $$\begin{aligned} \theta \leftarrow \theta - \eta \nabla _{\theta } \mathscr {J}, \end{aligned}$$

where $\theta$ represents the model parameters, $\eta$ is the learning rate, and $\nabla _{\theta } \mathscr {J}$ is the gradient of the objective function concerning the model parameters.

### Classification head

It plays a crucial role in the final stages of the model, aiming to perform the ultimate classification task after the contrastive learning step. In this phase, the latent space representations, carefully derived from the projection head, become pivotal. These representations are input into a fully connected layer, meticulously designed with four units, each corresponding to one of the four vehicle classes (bus, noise, Moto, or auto).

The fully connected layer is mathematically represented as follows:

5 $$\begin{aligned} \text {Class Scores} = W_{\text {class}} \cdot \text {Latent Space} + b_{\text {class}}, \end{aligned}$$

Here, $W_{\text {class}}$ signifies the weight matrix, $\text {Latent Space}$ represents the output of the projection head, and $b_{\text {class}}$ is the bias vector.

Subsequently, the Softmax Activation Function is applied to the obtained class scores:

6 $$\begin{aligned} \text {Class Probabilities} = \text {Softmax}(\text {Class Scores}) \end{aligned}$$

This activation function is pivotal in transforming raw scores into meaningful class probabilities. The classification head’s meticulous design and mathematical representation ensure the effective utilization of latent space representations for accurate and meaningful classification. The Softmax activation function, applied to the class scores, further refines the model’s predictions, converting them into interpretable class probabilities.

### Fine-tuning

Finally, the model undergoes fine-tuning on the labeled training data to adapt the learned features specifically for the seismic signal classification task. The fine-tuning Loss (Cross-Entropy) is given by:

7 $$\begin{aligned} L_{\text {fine-tune}}(x_i, y_i) = -\sum _{c=1}^{C} y_{i,c} \log (\text {Softmax}(W_{\text {class}} \cdot \text {Latent Space} + b_{\text {class}})), \end{aligned}$$

where $y_i$ is the one-hot encoded label for sample $x_i$, and $C$ is the number of classes.

The fine-tuning objective is formulated as:

8 $$\begin{aligned} \mathscr {J}_{\text {fine-tune}} = \frac{1}{N} \sum _{i=1}^{N} L_{\text {fine-tune}}(x_i, y_i) \end{aligned}$$

The overall fine-tuning objective is to minimize the average fine-tuning loss over the labeled data. The fine-tuning optimization is expressed as:

9 $$\begin{aligned} \theta \leftarrow \theta - \eta \nabla _{\theta } \mathscr {J}_{\text {fine-tune}}, \end{aligned}$$

Here, $\theta$ represents the model parameters, and they are updated using gradient descent during the fine-tuning process.

This process optimizes the model parameters further by minimizing the average fine-tuning loss. The comprehensive approach, encompassing self-supervised contrastive learning, data augmentation, and a carefully designed encoder network architecture, ensures accurate and efficient classification of seismic signals. The contrastive learning process facilitates the acquisition of meaningful feature representations. At the same time, the fine-tuning step tailors the model to the specific classification task, resulting in state-of-the-art performance on seismic signal classification tasks. This approach is a valuable and effective tool for various applications in seismic analysis and beyond.

## Results and discussion

Herein, we evaluate the performance of the proposed VC scheme. Table 2 details the dataset parameters conducted in the evaluation process. Also, Table  3 highlights the hyperparameter details of the compared methods (SVM,CNN, and LSTM), including our proposed CL approach. To promote a thorough assessment, the data was divided into three subsets: (20%) for training purposes, (60%) for validation, and (20%) for testing. This distribution guaranteed an equitable representation and dependable analysis. The testing set was an impartial evaluation of the model’s generalization ability. The performance evaluation used response time and VC accuracy as primary metrics. By employing these metrics, the system’s effectiveness was evaluated. Response time assesses the system’s velocity and effectiveness in producing outcomes, whereas VC accuracy evaluates the system’s capability to identify and categorize vehicles accurately. The obtained results from the self-supervised contrastive learning approach for vehicle seismic signal classification exhibit robustness and effectiveness, especially when combined with various data augmentation techniques.

**Table 2:** Parameters employed by default in the evaluation.

Parameter	Value
Data collection size	930
The training data size after augmentation	4650
The sampling rate of the data collection process	250 Hz
The Number of geophones	3
The distance from the geophones to the road	0.5 m

**Table 3:** Hyperparameter settings for investigated models.

Model	SVM	CNN	LSTM	Proposed CL
Batch size	N/A	32	64	128
Learning rate	N/A	0.001	0.0005	0.0003
Optimizer	N/A	Adam	RMSprop	AdamW
Loss function	Hinge loss	Cross-entropy	Cross-entropy	InfoNCE loss
Epochs	N/A	100	120	150
Dropout	N/A	0.5	0.3	0.2
Regularization	L2 (C = 1.0)	L2 (0.0001)	L2 (0.0005)	Weight decay (0.001)

**Fig. 10:**
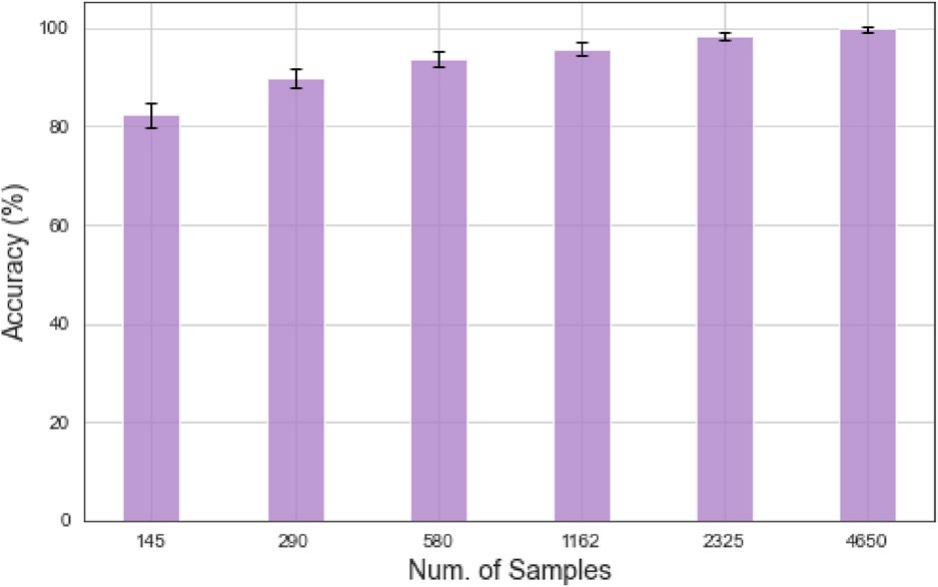
Accuracy comparison of different dataset sizes.

Figure 10 illustrates the model’s accuracy as the size of the training dataset increases. With a dataset size of 145 samples, the model achieves 82.4% accuracy. As the dataset size increases to 290 samples, the accuracy improves to 89.8%. Further increasing the dataset size to 580 samples leads to a significant improvement in accuracy, reaching 93.6%. With 1162 samples, the accuracy reaches 95.8%. The model achieves a high accuracy of 98.5% and 99.8% when the dataset size is increased to 2325 and 4650 samples, respectively. These results demonstrate the importance of having a more extensive training dataset to achieve higher model performance. As the dataset size increases, the model can learn more comprehensive representations and generalize better, improving accuracy.

**Fig. 11:**
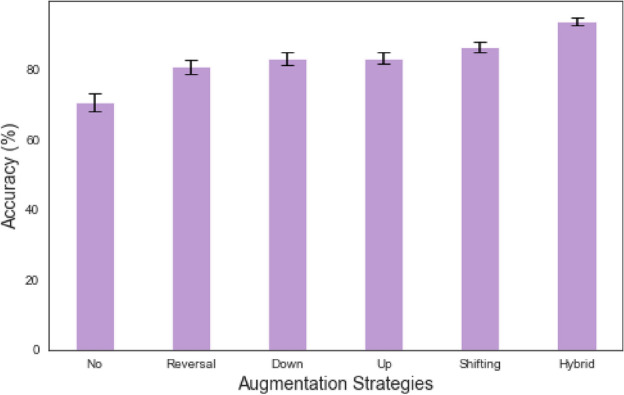
Accuracy comparison of different data augmentation methods on 580 samples.

Figure 11 compares the model’s accuracy using different data augmentation techniques on a dataset of 580 samples. Without any data augmentation, the model achieves an accuracy of 70.6%. Applying the “reversal” augmentation technique improves the accuracy to 80.8%. The “Down” and “Up” augmentation methods result in accuracies of 83.1% and 83.2%, respectively. The “Shifting” augmentation technique further boosts the accuracy to 86.3%. Combining multiple techniques, the “Hybrid” augmentation achieves the highest accuracy of 93.6%. These results demonstrate the effectiveness of data augmentation in improving the model’s performance, especially when the training dataset is relatively small. The “Hybrid” approach, which leverages multiple augmentation techniques, is the most beneficial in enhancing the model’s accuracy.

**Fig. 12:**
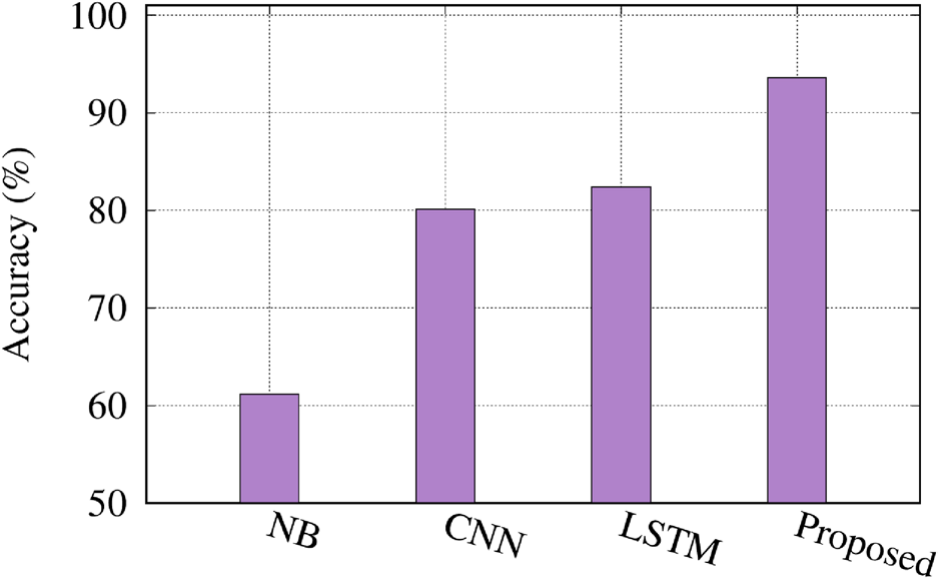
Accuracy comparison of the proposed approach and the latest technique on 580 samples.

Figure 12 compares the accuracy of the proposed approach and the latest technique on 580 samples. To ensure a fair comparison, we tested the same dataset using the previous algorithms with the identical hyperparameters they employed, i.e., convolutional neural networks (CNN), Naive Bayes (NB), Long Short-Term Memory (LSTM), as in^26,27,29^, respectively. The NB classifier^27^ achieves an accuracy of 61.2% on the dataset with 580 samples. The CNN model achieves an accuracy of 80.1% on the 580-sample dataset. The LSTM model^29^ achieves an accuracy of 82.4% on the 580-sample dataset. The proposed approach achieves the highest accuracy of 93.6% on the 580-sample dataset. The results demonstrate that the proposed approach significantly outperforms the latest techniques, including NB^27^, CNN^26^, and LSTM^29^, on the 580-sample dataset. This indicates that the proposed method can effectively utilize the available data and learn more comprehensive representations, leading to superior performance compared to the state-of-the-art models. The considerable gap between the accuracy of the proposed approach (93.6%) and the other techniques (61.2% for NB, 80.1% for CNN, and 82.4% for LSTM) highlights the effectiveness and robustness of the proposed method. This finding is particularly notable, as it demonstrates the proposed approach’s ability to achieve high accuracy even with a small dataset size of 580 samples. These results indicate that the proposed approach outperforms the latest techniques across all dataset sizes. This confirms the importance of data augmentation in improving the model’s ability to learn diverse and representative features. As the dataset size decreases (1162, 580, 290, and 145 samples), the performance of individual augmentation techniques gradually diminishes while the proposed hybrid approach remains more robust.

**Fig. 13:**
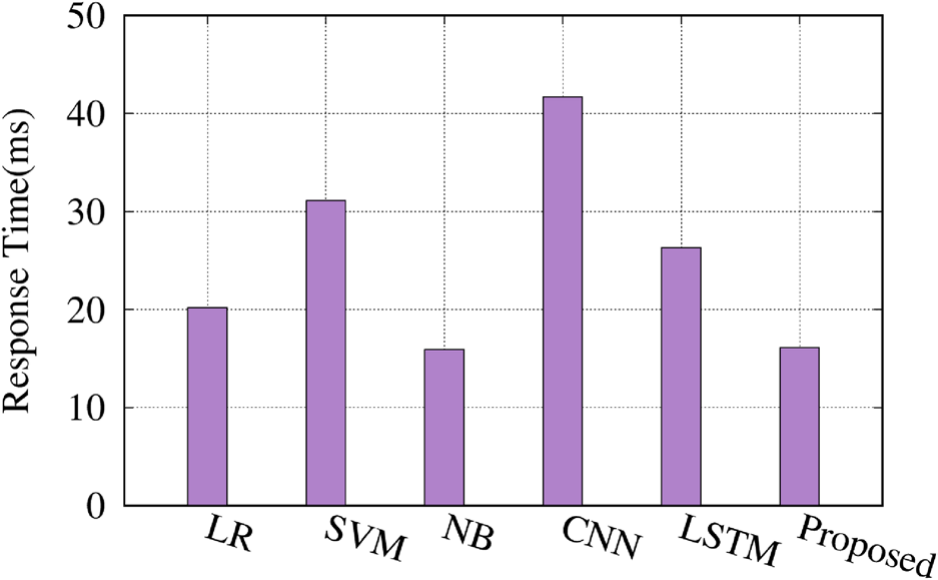
Comparison of the proposed approach with the latest techniques in terms of time.

Figure 13 compares the time taken by the proposed approach and the latest techniques. The NB classifier has the fastest inference time of 15.9 ms. The proposed approach takes 16.1 ms, slightly higher than NB but significantly faster than the other techniques. The SVM and LSTM models take 31.1 and 26.3 ms, respectively. The Logistic Regression (LR) and CNN models have the longest inference times of 20.2 and 41.7 s, respectively. These results demonstrate that the proposed approach balances accuracy and inference time well, making it a practical and efficient solution for real-world applications. The relatively fast inference time of the proposed approach is a desirable characteristic, especially in scenarios where quick decision-making is required. The results presented in Figs. 5, 6, 7 and 8 provide a comprehensive evaluation of the proposed approach and its performance compared to the latest techniques. The findings highlight the advantages of the proposed method in terms of accuracy, dataset efficiency, and inference time, making it a compelling choice for practical applications. The comprehensive results indicate that the self-supervised contrastive learning approach, especially with hybrid data augmentation, is highly effective for seismic signal classification. It consistently outperforms traditional classifiers, yielding state-of-the-art accuracy rates and demonstrating its potential for real-world applications in seismic analysis and related fields. Combining data augmentation and self-supervised learning is a powerful strategy for extracting meaningful and discriminative features from seismic signals, leading to superior classification performance across diverse dataset sizes. The self-supervised contrastive learning approach, in combination with hybrid data augmentation, presents a compelling solution for seismic signal classification tasks. The results indicate its effectiveness in extracting meaningful features from seismic signals and achieving state-of-the-art accuracy, especially in scenarios with limited labeled data.

**Table 4:** Performance metrics for fivefold cross-validation of the self-supervised contrastive learning model for vehicle classification using seismic data.

Fold	Accuracy (%)	Precision (%)	Recall (%)	F1-Score (%)	Contrastive loss
1	99.5	98.7	99.2	98.9	0.015
2	99.7	98.9	99.4	99.1	0.012
3	99.6	99.0	99.5	99.2	0.014
4	99.8	99.3	99.6	99.4	0.010
5	99.8	99.4	99.6	99.5	0.011

**Fig. 14:**
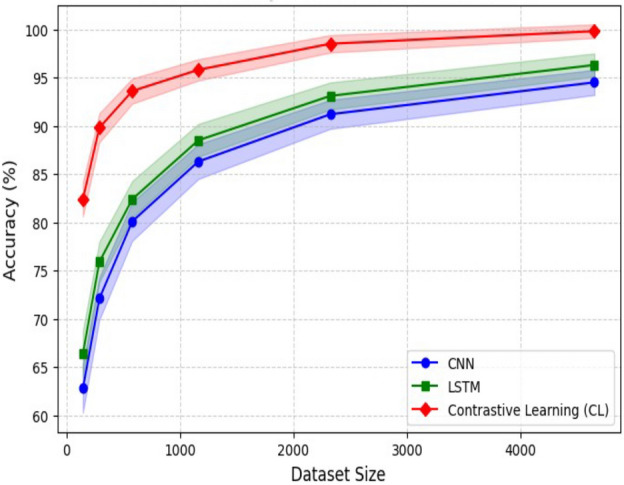
Model accuracy versus dataset size with 95% CL.

Table 4 summarizes the performance metrics obtained from the fivefold cross-validation for our proposed approach. The model consistently demonstrated high accuracy, precision, recall, and F1 scores, with minimal variation across the folds. Contrastive loss remained low throughout, indicating the model’s efficiency in separating seismic signal data from different vehicle classes. The results from the fivefold cross-validation clearly illustrate the model’s effectiveness and reliability in vehicle classification using seismic data. For further details, fivefold cross-validation divides the dataset into five equal parts or folds. The model is trained on four folds and tested on the remaining one. This process is repeated five times, with each fold acting as the test set once. This approach ensures the model performs well across different parts of the dataset, providing a more accurate assessment of its ability to generalize to unseen data. In this study, the model achieved impressive accuracy scores, ranging from 99.5 to 99.8% across all folds. This highlights its capability to classify different types of vehicles based on seismic signals, regardless of the test data. Such consistent accuracy suggests that the model is generalizing well and not overfitting to specific parts of the data. The data augmentation techniques used to expand the training dataset artificially were key to this success, ensuring more diverse and robust learning from seismic signals. These techniques helped prevent overfitting, enabling the model to perform well even with a limited dataset. The contrastive loss values remained low across all folds (between 0.010 and 0.015), a positive indicator of the model’s ability to differentiate between seismic signals from different vehicle classes. This is critical because lower loss values mean seismic signals from the same vehicle class are closely grouped. In contrast, signals from other classes are more distinctly separated, leading to higher classification accuracy. The high precision (up to 99.4%) and recall (up to 99.6%) further confirm the model’s effectiveness. High recall means that the model correctly identifies vehicles, while high precision shows that it avoids incorrect classifications. This balance is essential for traffic monitoring and accident prevention applications, where misclassifications can have serious consequences.

Figure 14 illustrates the relationship between dataset size and model accuracy, with a 95% confidence interval (CI) for each model. The results clearly demonstrate that CL consistently outperforms CNN and LSTM across all dataset sizes. CL achieves higher accuracy, showcasing its robust feature extraction capabilities. Even with a limited dataset of 145 samples, CL significantly outperforms CNN and LSTM, proving its effectiveness in data-scarce environments. As the dataset size increases, CL maintains its advantage, reaching near 100% accuracy at 4650 samples. CNN and LSTM exhibit gradual performance improvements as dataset size increases; however, their accuracy plateaus at lower levels than CL. This suggests that CNN and LSTM require larger datasets to enhance classification performance effectively. Additionally, all models show a steep accuracy increase when dataset size grows from 145 to 1162 samples, highlighting the importance of data availability. The confidence intervals indicate that CL exhibits higher stability (narrower CI), whereas CNN and LSTM have higher variance, mainly when working with smaller datasets. These findings reinforce the superiority of CL in seismic-based vehicle classification, demonstrating its strong generalization capabilities and making it an optimal choice for real-world deployment in ITSs.

**Table 5:** Pairwise t-test results (CL vs. others).

Comparison	t-statistic	*p* value	Significance
CL vs. SVM	32.1	< 0.0001	Significant
CL vs. CNN	28.5	< 0.0001	Significant
CL vs. LSTM	25.7	< 0.0001	Significant

**Fig. 15:**
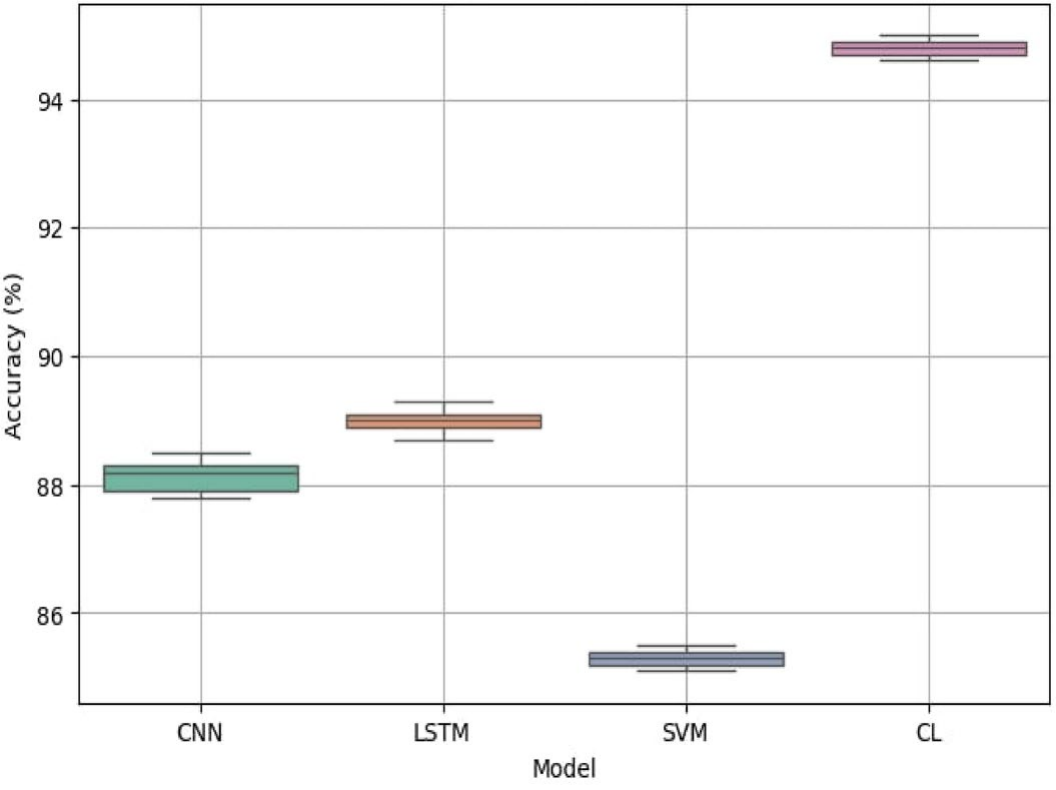
Model accuracy comparison ANOVA and pairwise t-tests.

The results presented in boxplot Fig. 15 and pairwise t-test Table 5 provide a comprehensive statistical evaluation of model performance differences in seismic-based vehicle classification. The ANOVA and boxplot analysis clearly indicate that CL achieves the highest accuracy compared to CNN, LSTM, and SVM. The boxplot visualization further highlights CL’s superior performance, demonstrating both higher accuracy and minimal variance, which confirms its stability across multiple runs. Additionally, the ANOVA test results (*p* value< 0.0001) validate that at least one model performs significantly differently from the others, reinforcing CL’s effectiveness. The pairwise t-test results, where all *p* values are< 0.0001, establish statistically significant performance differences among the models. The high t-statistics further confirm that CL substantially outperforms CNN, LSTM, and SVM in classification accuracy. While SVM is computationally efficient, its lack of deep feature extraction capabilities results in inferior performance, making it unsuitable for complex seismic-based vehicle classification tasks. Overall, CL’s significant advantage in classification accuracy, validated by ANOVA and pairwise t-tests, underscores its ability to learn highly discriminative features. The boxplot visualization reaffirms CL’s stable and consistent performance, highlighting its robustness in feature extraction and computational efficiency. These findings establish CL as the optimal model for real-time deployment in Intelligent Transportation Systems, ensuring both accuracy and efficiency in seismic-based vehicles.

The complexity analysis of the compared models, based on FLOPs and memory usage, are previewed in Tables 6 and 7, revealing significant differences in computational efficiency. SVM demonstrates the lowest computational cost with only 0.005 FLOPs and minimal memory usage (18 MB), making it the most lightweight model. However, its simplicity may come at the expense of performance in complex tasks. On the other hand, the CNN and LSTM models exhibit significantly higher FLOPs (2.3 and 4.7, respectively) and memory consumption (450 MB and 680 MB), indicating their substantial computational cost. LSTM, in particular, has the highest resource demand, which may limit its practical deployment in resource-constrained environments. Meanwhile, our proposed CL model achieves a balanced trade-off, requiring 1.9 FLOPs and 275 MB of memory. This optimized performance highlights its advantage in delivering competitive efficiency while maintaining a lower computational burden than CNN and LSTM. It is a more practical choice for real-world vehicle classification applications where accuracy, efficiency, and computational cost matter, especially since this system might be deployed on low-cost computers such as Raspberry Pi.

### Limitations

One limitation of this study is the absence of high-speed vehicle data. The experiments were conducted at Kyushu University’s ITO campus, where road regulations restrict vehicle speeds to 40 km/h. Therefore, the impact of high-speed vehicle transitions on seismic wave characteristics was not included and left for future investigation. However, since vehicle combustion engines generate a partial portion of the seismic waves, their frequency components are relatively independent of vehicle speed^41^. Nonetheless, higher-speed vehicles may introduce additional complexities, such as increased wave energy, possible Doppler effects, and variations in wave propagation patterns. Future studies should investigate these factors by collecting seismic data from high-speed vehicle environments to evaluate their impact on classification performance and model generalization.

Furthermore, our system is mainly designed to work in specific areas, such as one-lane streets, toll collections, intersections, etc. To accommodate any situation (multi-lane), we need to increase the number of deployed geophones on a large scale. Besides, our approach faces hardware limitations, such as geophone sensitivity and environmental interference, which can affect data accuracy. Additionally, software limitations include the need for significant computational resources for training and challenges in model generalization to diverse real-world traffic conditions, necessitating further fine-tuning and validation. Moreover, extreme environmental perturbations, such as heavy precipitation, can significantly affect seismic sensors by attenuating surface and S waves, potentially leading to system failures. While such perturbations are inherently challenging to mitigate, we propose several procedures to minimize their impact, including improved sensor shielding, adaptive noise filtering techniques, and site selection strategies that enhance measurement stability.

**Table 6:** Flop comparison of compared models.

Model	Flops	Efficiency rank
SVM	0.005	Fastest
CNN	2.3	High cost
LSTM	4.7	Very high cost
Contrastive learning	1.9	Optimized

**Table 7:** Memory usage comparison of compared models.

Model	Memory usage (MB)
SVM	18
CNN	450
LSTM	680
Contrastive learning	275

## Conclusion and future directions

This paper presents a novel vehicle classification technique that uses seismic data collected through geophones to improve intelligent transportation systems. The method addresses the limitations of traditional visual and sensor-based methods, which can be affected by harsh environmental conditions. The method is robust and privacy-preserving, making it a viable alternative for vehicle classification in ITSs. Key contributions include specialized data augmentation techniques, a self-supervised contrastive learning framework, and a detailed encoder network architecture and projection head architecture. Optimized with the Information Noise-Contrastive Estimation Loss function, the contrastive learning approach effectively clusters positive pairs while separating negative pairs in the latent space. The method achieves 99.8% accuracy in data-scarce scenarios, and its hybrid augmentation technique enhances the training dataset. Comparative evaluations against traditional classifiers consistently demonstrate the advantages of the proposed methodology. Our VC method adeptly manages complex traffic environments, categorizing vehicles into small, medium, and large classes. Individual classification is challenging, given the vast and ever-growing variety of vehicle models. However, our self-supervised contrastive learning approach, bolstered by robust data augmentation, ensures the model’s adaptability to diverse traffic conditions. The findings highlight this seismic data-based approach’s practical relevance and potential real-world applications, offering a cost-effective, privacy-preserving solution without compromising performance. Future research will focus on expanding data collection to include both seismic and conventional data (e.g., visual, radar) for comprehensive performance comparisons, exploring multimodal approaches by integrating seismic data with visual inputs to improve classification accuracy and robustness and fine-tuning and validating the model in diverse real-world traffic conditions with multiple-lane scenarios. Furthermore, we will enhance the resilience of seismic-based VC by developing adaptive calibration for sensor placement, integrating advanced noise filtering, and employing domain adaptation for robust signal processing. Additionally, multi-sensor fusion and transfer learning will be explored to improve model generalization, ensuring reliable performance across diverse road configurations and dynamic traffic conditions.

## Data Availability

The datasets generated during and analyzed during the current study are available from the corresponding author on reasonable request.
